# Aberrant Cluster of Differentiation (CD) Marker Expression Across B-cell Chronic Lymphoproliferative Disorder Subtypes: A Single-Center Flow Cytometry Study From Sri Lanka

**DOI:** 10.7759/cureus.117139

**Published:** 2026-09-29

**Authors:** Thushara Balasuriya, Chamarika Moonesinghe, Neluka Fernando, Chinthika Gunasekara, Surangi Jayakody, Chitranga Kariyawasan

**Affiliations:** 1 Hematology, Sri Jayewardenepura General Hospital, Colombo, LKA; 2 Hematology, University of Sri Jayewardenepura, Colombo, LKA; 3 Microbiology, University of Sri Jayewardenepura, Colombo, LKA; 4 Community Medicine, Warwick Medical School, University of Warwick, Coventry, GBR

**Keywords:** aberrant marker expression, chronic lymphocytic leukemia, flow cytometry, immunophenotype, mantle cell lymphoma

## Abstract

Introduction

Flow-cytometric immunophenotyping is central to the diagnosis and subclassification of B-cell chronic lymphoproliferative disorders (B-CLPDs). In routine practice, partial antigen loss, reduced expression, and unexpected antigen acquisition may create diagnostic difficulty, particularly when tissue-based or molecular confirmation is delayed. This study describes subtype-wise aberrant cluster of differentiation (CD) marker expression patterns in B-CLPDs diagnosed by integrated morphology and multiparameter flow cytometry in a Sri Lankan tertiary-care laboratory.

Methods

A descriptive cross-sectional analysis was performed on 210 diagnosed B-CLPD cases. Aberrancies were categorized as unexpected expression, antigen loss, or reduced expression using predefined subtype-specific expectations, internal reference populations, and routine laboratory fluorescence-intensity interpretation. Frequencies were reported as N (%) within each subtype, with denominators specified where relevant. Because several rare subtypes had small numbers, inferential testing was restricted to selected comparable aberrancy events between chronic lymphocytic leukemia (CLL) and mantle cell lymphoma (MCL).

Results

CLL was the commonest subtype, comprising 94 (44.8%) cases, followed by MCL with 34 (16.2%) and the CD5-negative/CD10-negative/hairy-marker-negative B-CLPD group with 31 (14.8%). In CLL, the most frequent events were unexpected CD20 expression in 44 (46.8%) cases, reduced CD45 in 42 (44.7%), unexpected surface kappa expression in 24 (25.5%), and reduced CD23 in 24 (25.5%). MCL showed reduced CD45 in 11 (32.4%) cases, reduced CD43 in eight (23.5%), reduced CD5 in four (11.7%), unexpected CD23 in four (11.8%), unexpected CD200 in four (11.8%), and reduced FMC7 in four (11.8%). The CD5-negative/CD10-negative/hairy-marker-negative B-CLPD group was the most heterogeneous, with reduced CD200 and reduced FMC7 each occurring in 15 (48.3%) cases.

Conclusion

CLL was characterized mainly by unexpected CD20 expression and reduced CD45, whereas MCL most commonly showed reduced CD45 and CD43. The CD5-negative/CD10-negative/hairy-marker-negative B-CLPD group showed the greatest immunophenotypic heterogeneity, particularly reduced CD200 and FMC7. These findings demonstrate that aberrant antigen expression is subtype-associated and should be interpreted within the complete immunophenotypic profile rather than using individual markers in isolation.

## Introduction

B-cell chronic lymphoproliferative disorders (B-CLPDs) are a heterogeneous group of mature B-cell neoplasms that may overlap clinically, morphologically, and immunophenotypically. Flow-cytometric immunophenotyping is one of the most useful first-line investigations because it rapidly establishes B-cell clonality, defines lineage, and identifies subtype-associated antigenic patterns. Current classification systems still require the integration of immunophenotype with morphology, clinical information, histology, and genetic findings when available [1,2].

Classical immunophenotypic patterns are well recognized. Chronic lymphocytic leukemia (CLL) usually shows cluster of differentiation 5 (CD5), CD23, and CD200 expression with weak surface immunoglobulin, CD20, and CD79b [2-5]. CD45 density has also been investigated as a useful discriminator in B-cell chronic lymphoproliferative disorders [6]. Mantle cell lymphoma (MCL) is typically CD5-positive, CD23-negative or dim, FMC7-positive, and CD79b-positive. CD43 can provide additional interpretive information in malignant flow cytometry assessment [7]. Recent multiparameter flow-cytometric scoring approaches have also been proposed for distinguishing typical CLL/small lymphocytic lymphoma (SLL), atypical CLL/SLL, and MCL [8,9]. CD5-negative/CD10-negative low-grade B-cell proliferations form a broad differential-diagnostic group that includes several overlapping mature B-cell neoplasms [10-13]. Classical hairy cell leukemia (HCL) usually expresses CD11c, CD25, CD103, and CD123, while HCL variant (HCL-v) has distinct clinicopathological and immunophenotypic features [14,15].

However, routine diagnostic practice frequently encounters reduced marker expression, antigen loss, or unexpected marker acquisition. These departures from canonical profiles may create difficulty, especially in the CLL-MCL differential diagnosis and within CD5-negative/CD10-negative low-grade B-cell proliferations [2-5,8,10-15].

A structured description of aberrant marker expression is useful for diagnostic laboratories. It identifies markers that remain stable within each subtype, highlights markers prone to attenuation, and points out situations where a single marker may mislead interpretation. This is particularly relevant in resource-limited settings, where flow cytometry may be the main available tool for initial subclassification while histology, immunohistochemistry, or molecular confirmation is pending or unavailable.

The aim of this study was to describe subtype-wise aberrant CD marker expression patterns in B-CLPDs diagnosed at a tertiary-care hematology laboratory in Sri Lanka.

## Materials and methods

Study design, setting, and cases

This descriptive cross-sectional study was conducted at the Department of Hematology of Sri Jayewardenepura General Hospital, Sri Lanka, from July 2023 to December 2025. The study included diagnosed B-CLPD cases confirmed by integrated clinicopathological assessment, including morphology, clinical findings, and multiparameter flow-cytometric immunophenotyping. Treated cases, inadequate samples, and cases with incomplete data were excluded. Ethical approval was obtained from the Ethics Review Committee of the University of Sri Jayewardenepura (approval number: ERC 08/23).

Sample size and sampling rationale

The sample size was determined to ensure adequate representation of B-CLPD cases for the planned descriptive analysis. The minimum sample size was calculated using the single-population proportion formula [16]:

$n = \frac{Z^{2} \times P(1-P)}{d^{2}}$

where n is the required sample size, Z is the critical value corresponding to a 95% confidence level (1.96), P is the expected population proportion, and d is the accepted absolute precision (0.05). The reported prevalence of 13.1% was approximated as P = 0.13, based on reported Sri Lankan non-Hodgkin lymphoma prevalence data [17]. The calculated minimum sample size was 174 cases; after adding 10% to account for nonresponse or incomplete data, the planned minimum sample size was 192. Consecutive eligible cases presenting during the study period were included, and recruitment was continued to maximize the representation of the less frequent B-CLPD subtypes. The final analysis therefore comprised 210 cases.

Flow cytometry and marker panel

Flow cytometry was performed on a BD FACSCanto II (BD Biosciences, San Jose, CA) using BD FACSDiva software version 8.0.1 (BD Biosciences, San Jose, CA). The panel included CD19 (PE Cy7), CD20 (V450), CD45 (V-500C), CD5 (PerCP Cy5.5), CD10 (PE), surface immunoglobulin (sIg) kappa (PE) and lambda (FITC), CD23 (FITC), CD79b (PerCP Cy5.5), CD200 (APC), CD38 (APC H7), CD43 (APC H7), and FMC7 (FITC). Hairy-cell-associated markers, including CD11c (PerCP Cy5.5), CD25 (FITC), CD103 (PE), and CD123 (APC), were used where clinically indicated. The panel and interpretation were aligned with WHO-based and published flow-cytometric diagnostic practice [1-3,8,10,14,15].

The gating strategy was performed in a stepwise manner. First, debris and nonviable events were excluded when applicable. The lymphocyte population was then identified using CD45 expression and side-scatter characteristics. B-cells were selected mainly using CD19 expression, and the relevant subtype-associated markers were assessed within the gated B-cell population.

Fluorescence-intensity data were generated on the same instrument using the same manufacturer-instructed protocol. Cytometer Setup and Tracking (CST) beads (BD Biosciences, San Jose, CA), BD OneFlow setup beads (BD Biosciences, San Jose, CA), BD OneFlow FC beads (BD Biosciences, San Jose, CA), and same-lot/same-conjugate reagents were used during the study period to reduce analytical variation. Because fluorescence intensity was not calibrated to Molecules of Equivalent Soluble Fluorochrome (MESF) or another external quantitative scale, the results should be interpreted as internally comparable laboratory findings rather than universal cut-off values.

Operational definition of aberrancy

Aberrancy was classified into three operational categories. Unexpected expression was defined as the detectable expression of a marker that is not usually expected in the assigned subtype. Antigen loss was defined as the complete absence of a marker that is usually expected to be present in that subtype. Reduced expression was defined as detectable but lower-than-expected fluorescence intensity compared with the usual subtype pattern, using internal reference populations, background nonneoplastic cells where appropriate, and routine laboratory interpretive standards on the same platform. Each aberrancy was assigned only after the review of the full immunophenotypic pattern, not from a single marker alone.

Statistical analysis

Statistical analysis in the parent study was performed using IBM SPSS Statistics version 20 (IBM Corp., Armonk, NY), and R 4.5.0 software (R Foundation for Statistical Computing, Vienna, Austria). Frequencies were summarized as N (%) within each subtype. Because several subtypes had small numbers and many marker events were subtype-specific, the main analysis was intentionally descriptive. For selected directly comparable aberrancy events between CLL and MCL, Fisher's exact test was used. Statistical findings were interpreted cautiously, and p-values were not used to make definitive diagnostic claims. The primary purpose of the analysis was to identify recurring subtype-wise patterns and practical diagnostic pitfalls.

## Results

Cohort distribution

The cohort included 210 B-CLPD cases. CLL was the dominant subtype, comprising 94 (44.8%) cases, followed by MCL with 34 (16.2%) and the CD5-negative/CD10-negative/hairy-marker-negative B-CLPD category with 31 (14.8%). Smaller groups included follicular lymphoma (FL), B-cell prolymphocytic leukemia (B-PLL), lymphoplasmacytic lymphoma (LPL), HCL, splenic marginal zone lymphoma (SMZL), atypical CLL, hairy cell leukemia variant (HCL-v), and diffuse large B-cell lymphoma (DLBCL). Therefore, the most reliable descriptive patterns came from CLL, MCL, and the CD5-/CD10-/hairy- group, while findings in rare subtypes should be regarded as exploratory. The subtype distribution of the study cohort is shown in Table 1.

**Table 1:** Distribution of B-CLPD subtypes included in the descriptive analysis Values are presented as N (%), where N is the exact number of cases and the percentage is calculated using the relevant cohort or subtype denominator CLL, chronic lymphocytic leukemia; MCL, mantle cell lymphoma; CD5-/CD10-/hairy- B-CLPD, CD5-negative/CD10-negative/hairy-marker-negative B-cell chronic lymphoproliferative disorder; FL, follicular lymphoma; B-PLL, B-cell prolymphocytic leukemia; LPL, lymphoplasmacytic lymphoma; HCL, hairy cell leukemia; SMZL, splenic marginal zone lymphoma; DLBCL, diffuse large B-cell lymphoma; HCL-v, hairy cell leukemia variant; N, number of cases

Subtype	N	%
CLL	94	44.8
MCL	34	16.2
CD5-/CD10-/hairy- B-CLPD	31	14.8
FL	9	4.3
B-PLL	8	3.8
LPL	7	3.3
HCL	7	3.3
SMZL	6	2.9
Atypical CLL	6	2.9
DLBCL	5	2.4
HCL-v	3	1.4

Subtype-wise aberrant marker patterns

In classical CLL, the main aberrancies were unexpected CD20 expression in 44 (46.8%) cases, reduced CD45 in 42 (44.7%), unexpected sIg kappa expression in 24 (25.5%), unexpected surface lambda expression in five (5.3%) CLL cases, and reduced CD23 in 24 (25.5%). Less frequent changes included unexpected CD79b in 18 (19.1%) cases, reduced CD5 in nine (9.5%), reduced CD200 in seven (7.4%), and unexpected FMC7 in seven (7.4%). These findings suggest marker attenuation and partial deviation within a still recognizable CLL pattern, rather than broad antigenic instability.

MCL showed a different profile, with reduced CD45 in 11 (32.4%) cases, reduced CD43 in eight (23.5%), reduced CD5 in four (11.7%), unexpected CD200 in four (11.8%), reduced FMC7 in four (11.8%), and unexpected CD23 in four (11.8%). This pattern not only supports separation from CLL but also shows that CD5 or CD43 attenuation does not exclude MCL when the full immunophenotypic pattern is supportive.

The CD5-/CD10-/hairy- B-CLPD category was the most heterogeneous group. Reduced CD200 and reduced FMC7 were each recorded in 15 (48.3%) cases, unexpected CD23 in 12 (38.7%), reduced CD45 in 11 (35.4%), unexpected CD38 in eight (25.8%), CD79b loss in seven (22.6%), and unexpected CD43 in five (16.1%). This broad spread supports the interpretation that this category represents a mixed non-CLL/non-MCL diagnostic compartment rather than one stable biological subtype.

Smaller subtypes broadly followed expected published patterns but were limited by small numbers. FL showed aberrancies involving CD19, CD45, CD23, and CD10. LPL showed limited aberrancy, including isolated unexpected CD5 and CD23. B-PLL largely retained its measured antigens apart from reduced CD45 in two (25.0%) cases. Classical HCL showed a stable phenotype with only reduced CD25 and CD123 in two (28.6%) cases each. HCL-v showed reduced CD200 in two (66.7%) of three cases. No recorded aberrancy was identified in the atypical CLL subgroup for the listed markers, and DLBCL showed only minor abnormalities. The main subtype-wise aberrant marker patterns are summarized in Table 2, and the selected recurrent events in the three largest groups are illustrated in Figure 1.

**Table 2:** Main aberrant marker patterns by subtype Each populated cell is presented as N (%), where N is the exact number of cases with the aberrancy and the percentage is calculated within that subtype CLL, chronic lymphocytic leukemia; MCL, mantle cell lymphoma; CD5-/CD10-/hairy- B-CLPD, CD5-negative/CD10-negative/hairy-marker-negative B-cell chronic lymphoproliferative disorder; FL, follicular lymphoma; LPL, lymphoplasmacytic lymphoma; B-PLL, B-cell prolymphocytic leukemia; HCL, hairy cell leukemia; HCL-v, hairy cell leukemia variant; DLBCL, diffuse large B-cell lymphoma; sIg, surface immunoglobulin; CD, cluster of differentiation; SMZL, splenic marginal zone lymphoma

Subtype	Aberrancy event	N	%
CLL	Reduced CD45	42	44.7
CLL	Unexpected CD20	44	46.8
CLL	Unexpected sIg kappa	24	25.5
CLL	Reduced CD23	24	25.5
CLL	Reduced CD5	9	9.5
CLL	Unexpected CD79b	18	19.1
CLL	Reduced CD200	7	7.4
CLL	Unexpected FMC7	7	7.4
CLL	No aberrant event recorded for listed markers	6	6.4
MCL	Reduced CD5	4	11.7
MCL	Reduced CD43	8	23.5
MCL	Reduced CD45	11	32.4
MCL	Unexpected CD200	4	11.8
MCL	Reduced FMC7	4	11.8
MCL	Unexpected CD23	4	11.8
MCL	No aberrant event recorded for listed markers	6	17.6
CD5-/CD10-/hairy- B-CLPD	Unexpected CD38	8	25.8
CD5-/CD10-/hairy- B-CLPD	Reduced CD45	11	35.4
CD5-/CD10-/hairy- B-CLPD	Reduced CD200	15	48.3
CD5-/CD10-/hairy- B-CLPD	CD79b loss	7	22.6
CD5-/CD10-/hairy- B-CLPD	Unexpected CD43	5	16.1
CD5-/CD10-/hairy- B-CLPD	Unexpected CD23	12	38.7
CD5-/CD10-/hairy- B-CLPD	Reduced FMC7	15	48.3
CD5-/CD10-/hairy- B-CLPD	No aberrant event recorded for listed markers	2	6.5
FL	Reduced CD10	1	11.1
FL	Reduced CD45	3	33.3
FL	Reduced CD19	4	44.4
FL	Unexpected CD23	3	33.3
FL	No aberrant event recorded for listed markers	1	11.1
LPL	Reduced CD45	3	42.9
LPL	Unexpected CD5	1	14.3
LPL	Unexpected CD23	1	14.3
LPL	No aberrant event recorded for listed markers	1	14.3
B-PLL	Reduced CD45	2	25
B-PLL	No aberrant event recorded for listed markers	6	75
HCL	Reduced CD25	2	28.6
HCL	Reduced CD123	2	28.6
HCL	No aberrant event recorded for listed markers	2	28.6
HCL-v	Reduced CD200	2	66.7
HCL-v	No aberrant event recorded for listed markers	0	0
SMZL	No aberrant event recorded for listed markers	1	16.7
Atypical CLL	No aberrant event recorded for listed markers	6	100
DLBCL	Reduced CD10	1	20
DLBCL	Reduced CD45	1	20
DLBCL	Reduced CD200	1	20
DLBCL	No aberrant event recorded for listed markers	1	20

**Figure 1:**
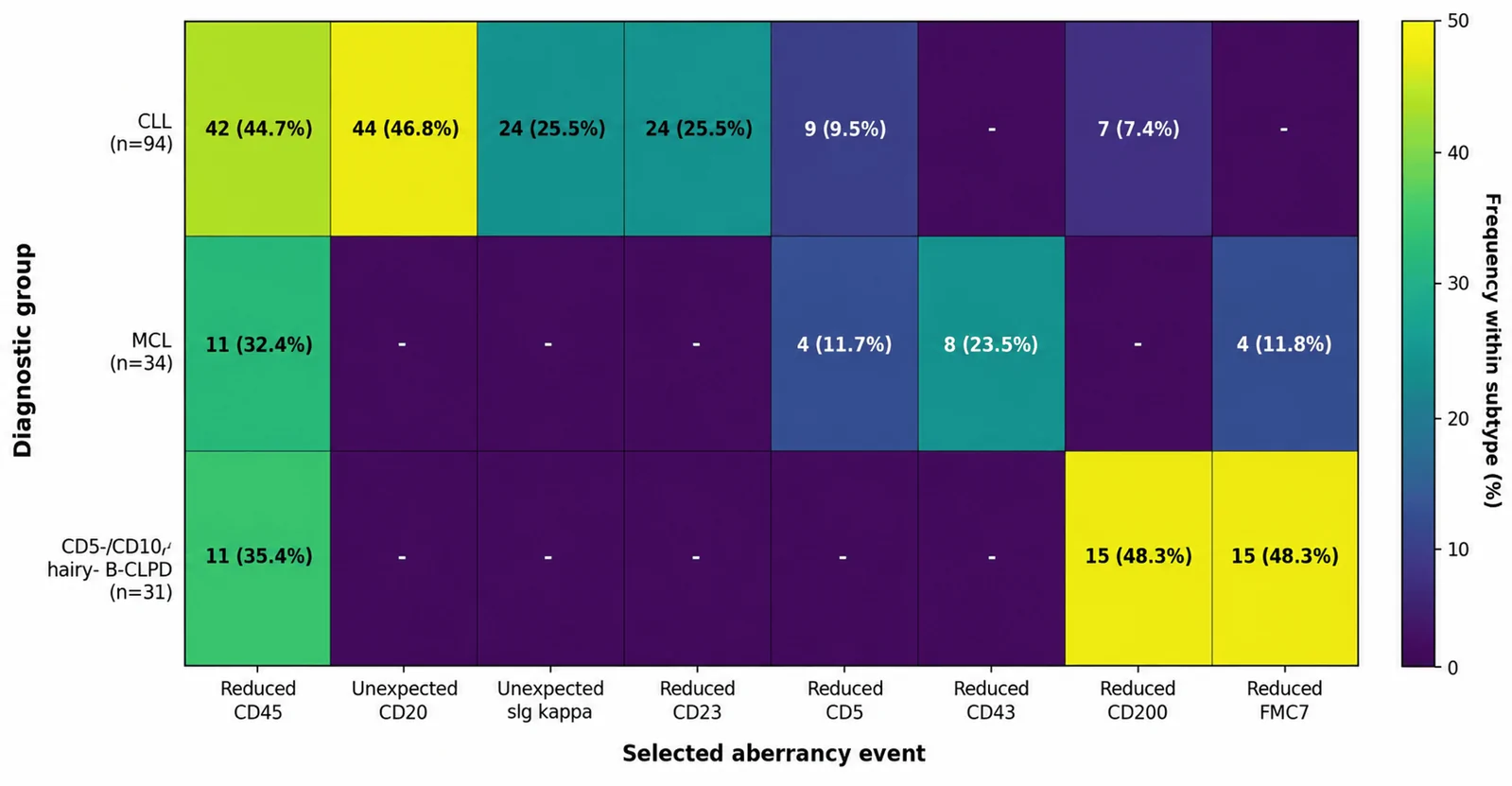
Selected aberrant CD marker patterns in the three largest B-CLPD groups The heat map summarizes selected recurrent aberrancy events in CLL, MCL, and the CD5-negative/CD10-negative/hairy-marker-negative B-CLPD group. Each populated cell is presented as N (%), where N is the exact number of cases with the aberrancy and the percentage is calculated within that subtype. Dashes ("-") indicate that no aberrancy was reported for that marker in the respective subtype B-CLPD, B-cell chronic lymphoproliferative disorder; CLL, chronic lymphocytic leukemia; MCL, mantle cell lymphoma; CD, cluster of differentiation; sIg, surface immunoglobulin; n, number of cases

Selected CLL-MCL comparisons

Only directly comparable aberrancy events were tested inferentially. Reduced CD45 was more frequent in CLL than MCL numerically, but the difference was not statistically significant in this dataset. Reduced CD5 was also not significantly different between the two groups. These results reinforce that single-marker aberrancies should not be used alone to separate CLL and MCL. The reduced CD45 events between CLL and MCL are shown in Table 3.

**Table 3:** Selected comparable aberrancy events between CLL and MCL Frequencies are presented in separate N and % columns. Values are presented as N (%), with percentages calculated within each diagnostic group. P-values were calculated using Fisher's exact test CLL, chronic lymphocytic leukemia; MCL, mantle cell lymphoma; n, number of cases with the event; CD, cluster of differentiation

Aberrancy event	CLL, N	CLL, %	MCL, N	MCL, %	Odds ratio	Fisher's exact p-value
Reduced CD45	42	44.7	11	32.4	1.69	0.23
Reduced CD5	9	9.5	4	11.7	0.79	0.744

## Discussion

This study shows that aberrant CD marker expression in B-CLPDs is not random. The patterns were subtype-related and remained interpretable when assessed in the context of the full immunophenotypic profile. CLL and MCL retained recognizable but modified profiles, while the CD5-/CD10-/hairy- group showed the greatest heterogeneity. This supports the practical principle that B-CLPD diagnosis should be based on overall antigenic architecture rather than isolated marker expression [1-3,8].

The CLL findings are consistent with established flow-cytometric knowledge. Classical CLL is typically defined by CD5, CD23, and CD200 expression with weak surface immunoglobulin and weaker expression of mature B-cell markers such as CD20 and CD79b [2-5,8,9]. In this context, unexpected CD20 expression, reduced CD45, surface light-chain-related aberrancy, reduced CD23, and unexpected CD79b are best interpreted as the modulation of antigen density within a recognizable CLL phenotype. This is diagnostically important because such cases may appear less classical if each marker is considered separately.

MCL also retained its expected diagnostic identity, but reduced CD5, reduced CD43, reduced CD45, unexpected CD23, unexpected CD200, and reduced FMC7 were observed in a minority of cases. These findings reinforce that the attenuation of CD5 or CD43 does not exclude MCL. Overreliance on one expected marker may lead to misclassification, especially when morphology is borderline or when confirmatory tests such as cyclin D1, SOX11, or t(11;14) are delayed [2,7,8].

The CD5-/CD10-/hairy- B-CLPD group was the most diagnostically mixed compartment. This result is expected because CD5-negative/CD10-negative small B-cell proliferations include several overlapping entities, such as splenic marginal zone lymphoma, lymphoplasmacytic lymphoma, and other low-grade splenic or leukemic B-cell neoplasms [10-13]. In such cases, flow cytometry is very useful for narrowing the differential diagnosis, but final classification often requires clinicopathological correlation, histology, and genetic findings when available.

The HCL-related findings also support the value of pattern-based interpretation. Classical HCL was relatively stable, with preserved canonical marker expression and only minor reduction in CD25 and CD123. HCL-v showed reduced CD200 in two (66.7%) of three cases, in keeping with the known divergence of HCL-v from classic HCL [14,15]. However, because these subgroups were small, these findings should be regarded as descriptive observations only.

The main practical value of this study is the local subtype-wise summary of aberrant marker expression from a Sri Lankan tertiary-care laboratory. In settings where advanced confirmatory tests may not be immediately available, the careful recognition of recurrent aberrancy patterns can help laboratories avoid overinterpreting single-marker deviations. The results also provide a baseline for future regional studies using standardized fluorescence-intensity thresholds and multicenter validation.

Limitations

This was a single-center study, and several subtypes had small sample sizes. Therefore, findings for rare groups such as HCL-v, DLBCL, atypical CLL, SMZL, LPL, and B-PLL should be considered exploratory.

Reduced marker expression was defined using internal reference populations and routine laboratory interpretive standards rather than externally calibrated Molecules of Equivalent Soluble Fluorochrome (MESF) values. This limits the direct transferability of intensity-related observations to other laboratories.

Molecular or immunohistochemical confirmation, including cyclin D1, SOX11, and t(11;14) for all suspected MCL cases, was not available for correlation in all cases.

The study was mainly descriptive. Broader inferential statistics or diagnostic cut-off modelling would require larger samples, standardized intensity calibration, and external validation.

## Conclusions

In this 210-case B-CLPD cohort, CLL was most commonly associated with unexpected CD20 expression in 44 (46.8%) cases and reduced CD45 in 42 (44.7%), whereas MCL most often showed reduced CD45 in 11 (32.4%) cases and reduced CD43 in eight (23.5%). The CD5-/CD10-/hairy- B-CLPD group showed the greatest heterogeneity, with reduced CD200 and reduced FMC7 each present in 15 (48.3%) cases and unexpected CD23 in 12 (38.7%). Reduced CD45 and reduced CD5 did not differ significantly between CLL and MCL, indicating that these individual aberrancies are insufficient for subtype distinction. Overall, the findings show that aberrant antigen expression is subtype-associated but must be interpreted within the complete immunophenotypic pattern, together with morphology and other clinicopathological findings.
